# Effect of Dietary Papaya (*Carica papaya*) Leaf Meal on Growth Performance and Haematological Responses of Nile Tilapia (*Oreochromis niloticus*) Fingerlings Reared in a Recirculating Aquaculture System

**DOI:** 10.1002/vms3.71271

**Published:** 2026-10-15

**Authors:** Farhabun Binte Farhad, Farzana Yasmin Ritu, Moupia Mustafi, Md. Abdus Salam, Shaharior Hashem

**Affiliations:** ^1^ Department of Aquaculture Bangladesh Agricultural University Mymensingh Bangladesh

**Keywords:** feed ingredient, growth promoter, haematological parameter, Nile tilapia, soybean‐meal replacement

## Abstract

In the pursuit of sustainable aquaculture, the integration of unconventional plant‐based feed additives like papaya leaf meal (PLM) offers a promising approach for enhancing fish growth while reducing feed costs and environmental impact. To evaluate the efficacy of PLM as a growth promoter, four experimental diets were formulated by gradually incorporating PLM at inclusion levels of 0%, 3%, 6% and 9%. Nile tilapia fingerlings were cultured over a 60‐day period within a recirculating aquaculture system comprising 12 tanks, with three replicates assigned to each treatment group. Fish were fed twice daily at a rate corresponding to 3% of their body weight. The diet with the highest inclusion of PLM (9%) resulted in significantly (*p* < 0.05) improved mean weight gain (11.44 g), per cent weight gain (82.67%), specific growth rate (SGR) (1.06%/day), feed conversion ratio (2.23), feed conversion efficiency (0.44), protein efficiency ratio (1.28) and overall fish yield (2.04 kg/m^3^/60 days) when compared to the control, 3% and 6% PLM‐based diet. Furthermore, the length–weight relationship in all dietary treatments indicated a positive allometric growth pattern in Nile tilapia fingerlings. A notable improvement in haematological parameters, red blood cells (3.10 ± 0.95 × 10^6^/mm^3^), white blood cells (132.98 ± 2.26 × 10^3^/mm^3^) and haemoglobin (12.15 ± 0.68 g/dL) was observed in treatment having 9% PLM with statistically significant differences among the treatments (*p* < 0.05). Therefore, these findings indicate that PLM inclusion up to 9% in diet is non‐toxic and positively impacts growth performance, making it a viable growth enhancer in tilapia fingerling diets.

## Graphical abstract

The study examined the efficacy of papaya leaf meal as a growth promoter of Nile tilapia fingerling. Certain level (9%) of papaya leaf meal in diet can improve the weight gain, SGR, PER and FCR of fish. It can also enhance the haematological parameters such as RBC, WBC and haemoglobin content of tilapia fingerling.

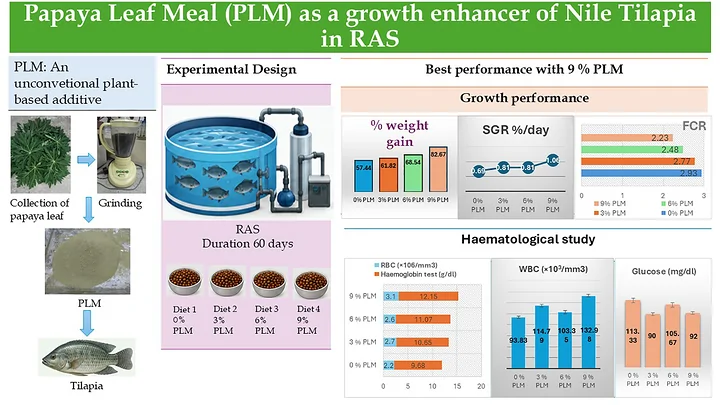

## 1 Introduction

Bangladesh's aquaculture sector has driven rapid agro‐food growth, more than doubling its production over the past decade to an estimated ∼2.6–3.0 million tonnes (MT) in 2021, contributing to overall fisheries expansion and food security (FAO 2023). Aquaculture has tremendous potential to help feed and nourish the world's growing population, but its growth must be managed sustainably (FAO 2022). The growing need for sustainable aquaculture has led to a great deal of research into substitutes for conventional fish feed ingredients (fish meal and fish oil), which are associated with economic and environmental problems (Rosle et al. 2024). The cost of commercial feed is increasing daily operational expenses, negatively affecting small‐scale farmers’ revenue. Reducing fish growth time through improved growth strategies could benefit the aquaculture industry, particularly for small‐scale farmers (Hamid et al. 2022).

Feed additives in aquaculture enhance nutrient utilization, growth and overall health. Aquatic feed is enriched with dietary supplements to enhance nutrient absorption (Dawood et al. 2021; Zheng et al. 2020) and support overall fish health (Shekarabi et al. 2022). Exogenous enzymes, amino acids and beneficial microbes are key feed additives that promote fish growth (Kari et al. 2022; Mohammadi et al. 2022; Cerqueira et al. 2020; Encarnação 2016; Castillo and Gatlin 2015). Among them, proteolytic enzymes are increasingly used in fish feed due to the rapid expansion of aquaculture and their cost‐effective benefits. These enzymes, derived from animals, plants or microorganisms (Zulhisyam et al. 2020; Agyei et al. 2019; Singh et al. 2019; Walsh et al. 1993), help improve digestion and nutrient absorption. Proper incorporation of exogenous enzymes can compensate for endogenous enzyme deficiencies and enhance growth performance (Zheng et al. 2020; Encarnação 2016). Plant‐based additives have gained popularity due to their bioactive compounds, such as alkaloids and polyphenols, which stimulate appetite, boost growth and strengthen immunity (Zhao et al. 2015). They are also cost‐effective, widely available and eco‐friendly.

Papaya (*Carica papaya*) is an herbaceous fruiting plant from the family Caricaceae (Kokila et al. 2016) and is its most widely cultivated and economically important member. Native to Central America and southern Mexico, it is now grown across tropical and subtropical regions worldwide (Orzechowski et al. 2002). *C. papaya*, known for its papain and chymopapain enzymes, possesses antiviral, antibacterial and antifungal properties (Aravind et al. 2013). The enzyme is essential to many biological processes in physiological and pathological states, medication designs and industrial applications, including meat tenderizers and pharmaceutical preparations because it can break down organic polymers formed of amino acids known as polypeptides (Mamboya and Amri 2012). Papaya leaves, often discarded as waste, contain bioactive compounds like flavonoids, alkaloids, vitamins and papain that have the potential to be a sustainable fish growth promoter in aquafeed. They are commonly used as feed additives in poultry, livestock and aquaculture, and studies show that papaya leaf extracts enhance fish growth and nutrient utilization (Pandey et al. 2016). Papaya plant produces a proteolytic enzyme called papain which is known to have various fish health benefits (Mic Salomiya et al. 2023). Research has shown that incorporating papain into aquafeed enhances growth performance and optimizes feed utilization in several fish species, such as sterlet (*Acipenser ruthenus*) (Wiszniewski et al. 2022), African catfish (*Clarias gariepinus*) (Rachmawati et al. 2020), mahseer (*Tor tambra*) (Muchlisin et al. 2016) and rohu (*Labeo rohita*) (Khati et al. 2015).

Recirculating aquaculture systems (RAS) are land‐based, engineered fish production systems that continuously treat and reuse water, significantly reducing water consumption compared with traditional systems (Shahbaziet al. 2025). By providing a controlled environment with stable water quality, RAS can reduce stress and promote growth and health in cultured fish (Ahmed and Turchini 2021). The system relies on nitrifying bacteria, such as *Nitrosomonas* and *Nitrobacter*, to convert ammonia produced by fish metabolism into less toxic forms, thereby recycling nutrients (Timmons and Ebeling 2010). RAS also regulates key culture conditions, including water flow, stocking density, temperature and chemical parameters, while efficiently removing metabolic waste such as faeces, ammonia, carbon dioxide and residual feed (Murray et al. 2014). Overall, these features create an optimal environment for fish physiological balance and growth.

Nile tilapia (*Oreochromis niloticus*) is globally farmed as an affordable, high‐quality protein source (Canonico et al. 2005), including minerals like iron, zinc, potassium, calcium, magnesium and sodium (Job et al. 2015), with Bangladesh ranking third in Asia for production (DoF 2023). Its popularity among small‐scale farmers is due to its reasonable operational costs, particularly for feed (Mohammadi et al. 2022). As an omnivorous fish, tilapia has lower protein needs compared to carnivorous species and can thrive on a variety of food resources (Ringø 2020). Its adaptability to different environmental conditions, such as salinity, oxygen levels and overcrowding, makes tilapia ideal for aquaculture (Coward and Bromage 2000). Due to its high market demand, ease of production and ability to adapt to the local climate, tilapia is currently one of the most popular aquaculture species in Bangladesh.

Previously, the use of papaya leaf extract in fish mainly focused on its microbicidal properties against fish pathogens (Azizah and Fasya 2019), whereas limited reports are available on the potential of papaya leaf meal (PLM) as a dietary growth supplement in aquafeed. In particular, the effects of whole PLM on the growth performance and haematological parameters of Nile tilapia remain insufficiently documented. Thus, this study aims to investigate the effect of PLM on the growth performance and haematological parameters of Nile tilapia fingerlings and also ensure the potentiality to be used as an alternative plant‐based protein source by fish farmers.

## 2 Materials and Methods

### 2.1 Experimental Site and Fish

The study was implemented at the Aquaponics Laboratory, Department of Aquaculture spanning a 60‐day period from November 2024 to January 2025. The study involved three replications for each treatment. Twelve fish tanks, each holding 200 L of water, were used, with gravel‐based media supporting the water recirculation system. The Nile tilapia fingerlings were obtained and kept in acclimatized conditions for 7 days before stocking in the experimental containers. Following acclimatization, each of the tanks was provided with the same biomass of fish (around 400 g). The initial length and weight of the fingerlings were 8.47 ± 0.27 cm and 14.79 ± 0.70 g, respectively.

### 2.2 Experimental Design

Twelve plastic water tanks, each measuring 0.22 m^3^ and containing 300 L, were utilized for fish culture and fingerling rearing. Approximately 200 L of clean water was added to each tank after it had been cleaned and washed. Each tank had a 20‐W submersible pump installed to circulate wastewater from the fish tank to the biological filter. Each tank was equipped with an 18‐W air pump and two stiff, perforated air stones to continuously deliver dissolved oxygen to the fish and prevent oxygen shortages for 24 h. The treatments were randomly assigned to the twelve tanks following a completely randomized design (CRD) to ensure the unbiased distribution and to have reliable results. The treatments involved papaya leaf at levels of 0%, 3%, 6%, and 9%, which were labelled as T_0_, T_1_, T_2_ and T_3_, respectively. Each tank was considered an independent experimental unit, with three tanks serving as biological replicates for each treatment (*n* = 3). Fish were fed twice a day, at 9:00 AM and 5:00 PM, with the amount set at 3% of their body weight. Fish were randomly sampled every 15 days using a scoop net. Various water quality parameters were monitored using API AquaTest kits.

### 2.3 PLM Preparation and Diet Formulation

Papaya leaves were freshly harvested from trees located along the southern pond banks of the experiment site. Following collection, the leaves were separated from their branches and dried using a homemade drier at 40°C for 2 days. Once dried, they were weighed and ground into a fine powder, which was then incorporated into feed preparation. The proximate composition of the PLM was analysed in the Fish Nutrition Laboratory following the standard protocol of AOAC.

The diet formulation included fish meal, soybean meal, wheat bran, rice bran, mustard oil cake, vitamins and PLM. The proximate composition of these ingredients was analysed and is detailed in Table 1. PLM was incorporated at 0, 3, 6 and 9 g, and the diets were designed to provide an iso‐nitrogenous diet containing 35% crude protein content, following Pearson's square method (Table 2). The ingredients were carefully mixed, with molasses as a binder, and then processed into 2 mm pellets using a pelletizer. After palletization, the feeds were sun‐dried for 2 days and stored in airtight containers at 20°C temperature.

**TABLE 1:** Proximate composition of papaya leaf and fish feed ingredients.

Nutrients composition (%)	Papaya leaf	Fish meal	Soybean meal	Rice bran	Mustard oil cake	Wheat bran
Moisture	23.21	10.82	17.07	13.83	15.68	14.25
Crude lipid	16.16	6.44	3.33	4.23	4.23	3.84
Crude protein	25.25	54.7	33.67	9.13	30.15	13.67
Crude fibre	22.53	2.21	5.21	8.74	4.83	6.77
Ash	11.2	9.3	6.49	11.85	10.7	7.79
Carbohydrate	24.86	16.53	34.34	52.22	34.41	53.68

**TABLE 2:** Percentage inclusion rates of ingredients for feed formulation.

Ingredients	Treatments			
	T_0_ 0% PLM	T_1_ 3% PLM	T_2_ 6% PLM	T_3_ 9% PLM
Fish meal	33	33	33	33
Soybean meal	33	30	27	24
Papaya leaf meal	0	3	6	9
Wheat bran	8.5	8.5	8.5	8.5
Rice bran	8.5	8.5	8.5	8.5
Mustard oil cake	15	15	15	15
Molasses	1	1	1	1
Vitamin	1	1	1	1
Total	100	100	100	100

### 2.4 Assessment of Fish Growth Parameters

Fish growth in terms of total length and body weight was measured at 15‐day intervals throughout the experimental period. At the end of the study, fish growth parameters were determined and calculated as follows:

$$\mathrm{Weight}\;\mathrm{gain},\;\mathrm{WG}\left(g\right)=\mathrm{final}\;\mathrm{weight}\left(g\right)-\mathrm{initial}\;\mathrm{weight}\left(g\right)$$

$$\mathrm{Per}\;\mathrm{cent}\;\mathrm{weight}\;\mathrm{gain},\;\mathrm{PWG}\;\left(\%\right)=\frac{\mathrm{weight}\;\mathrm{gain}\left(g\right)}{\mathrm{initial}\;\mathrm{weight}\left(g\right)}\times 100$$

$$\begin{array}{ccc} & & \mathrm{Specific}\;\mathrm{growth}\;\mathrm{rate},\;\mathrm{SGR}\;\left(\%\;\mathrm{per}\;\mathrm{day}\right)\\ & & \;=\frac{\ln\left[\mathrm{final}\;\mathrm{weight}\left(g\right)\right]-\ln\left[\mathrm{initial}\;\mathrm{weight}\left(g\right)\right]}{\mathrm{no}.\;\mathrm{of}\;\mathrm{days}\;}\times 100\; \end{array}$$

$$\mathrm{Food}\;\mathrm{conversion}\;\mathrm{ratio},\;\mathrm{FCR}=\frac{\mathrm{total}\;\mathrm{feed}\;\mathrm{intake}\left(g\right)}{\mathrm{total}\;\mathrm{wet}\;\mathrm{weight}\;\mathrm{gained}\left(g\right)}$$

$$\mathrm{Feed}\;\mathrm{conversion}\;\mathrm{efficiency},\;\mathrm{FCE}=\frac{\mathrm{Biomass}\left(g\right)\;}{\mathrm{total}\;\mathrm{feed}\;\mathrm{intake}\left(g\right)\;}$$

$$\mathrm{Protein}\;\mathrm{efficiency}\;\mathrm{ratio},\;\mathrm{PER}=\frac{\;\mathrm{weight}\;\mathrm{gained}\left(g\right)\;}{\mathrm{crude}\;\mathrm{protein}\;\mathrm{fed}\;\left(g\right)\;}$$

$$\begin{array}{ccc} & & \mathrm{Survival}\;\mathrm{rate},\;\mathrm{SR}\;\left(\%\right)\\ & & \;=\frac{\left(\mathrm{Initial}\;\mathrm{number}\;\mathrm{of}\;\mathrm{fish}\;\mathrm{stocked}-\mathrm{mortality}\right)}{\mathrm{Initial}\;\mathrm{number}\;\mathrm{of}\;\mathrm{fish}\;}\times 100\; \end{array}$$

The length–weight relationship of the experimental fish length–weight (L–W) relationship was analysed by the following formula:

$$W=qL^{b}$$

where *W* is the weight of the fish (in grams), *L* is the observed total length (in centimetres), *q* is the regression intercept, *Y* represents the dependent variable (logarithm of weight), *x* is independent variable (logarithm of length), *a* is the intercept of the regression line (logarithm of constant), and *b* is the regression slope, which is approximately three for isometric growth. When the equation is transformed logarithmically, it becomes

$$\log\;W=\log\;q+b\;\left(\log\;L\right)$$

This form is equivalent to a linear equation:

Y=bx+a

### 2.5 Assessment of Water Quality Parameters

Water quality parameters, such as temperature, pH, ammonia (NH_3_), nitrite (NO_2_), nitrate (NO_3_), total dissolved solids (TDS) and electrical conductivity (EC), were regularly monitored. Temperature, EC and TDS were measured using an EC meter, whereas pH, NH_3_, NO_2_ and NO_3_ concentrations were analysed through Sera test kits, sera GmbH, D 52518 (made in Germany) following standard procedures. All measurements were conducted at 15‐day intervals throughout the experimental period.

### 2.6 Assessment of Blood and Haematopoietic Indicators

Haematological parameters such as red blood cells (RBCs), white blood cells (WBCs), haemoglobin (Hb), glucose (Glc) and mean corpuscular haemoglobin (MCH) were analysed after 60 days of rearing. Blood samples were collected by severing the caudal peduncle of the experimental fish and drawing blood using a micropipette. To prevent coagulation, blood was extracted with a haematocrit tube, following the method of Hesser (1960). The samples were then transferred to Eppendorf tubes and refrigerated at 4°C for preservation. RBC and WBC counts were performed using a Neubauer Haemocytometer under a light microscope. Haemoglobin levels were measured using the cyanmethaemoglobin method, as described by Blaxhall and Daisley (1973). The RBCs, WBCs and MCH values were calculated using the following formulas:

$$\mathrm{RBC}\;\mathrm{count}=\frac{\mathrm{Number}\;\mathrm{of}\;\mathrm{large}\;\mathrm{square}\;\mathrm{cell}\;\left(5\right)\times \mathrm{Dilution}\;\mathrm{factor}\;\left(200\right)\times \mathrm{Counting}\;\mathrm{factor}\;\left(4000\right)\;}{\mathrm{Number}\;\mathrm{of}\;\mathrm{small}\;\mathrm{squares}\;\mathrm{counted}\;\left(80\right)}$$

$$\mathrm{WBC}\;\mathrm{count}=\frac{\mathrm{Number}\;\mathrm{of}\;\mathrm{large}\;\mathrm{square}\;\mathrm{cell}\;\left(1\right)\times \mathrm{Dilution}\;\mathrm{factor}\left(40\right)\;}{\;\mathrm{Volume}\;\mathrm{factor}\left(0.1\right)}$$

$$\mathrm{MCH}\;\left(\mathrm{pg}\right)=\frac{\mathrm{Hb}\times 10}{\mathrm{RBC}}$$

### 2.7 Statistical Analysis

Statistical evaluation of the data was conducted using one‐way analysis of variance (ANOVA). To identify significant differences among treatment groups, Duncan's Multiple Range Test was applied, considering a significant level of *p* < 0.05. All statistical analyses were performed using IBM SPSS Statistics 25.0 software.

## 3 Results

### 3.1 Evaluation of Feed Nutrients and Quality

The proximate analysis of PLM revealed a composition of 25.25% crude protein and 16.16% crude lipid, with moisture, crude fibre, ash and carbohydrate contents recorded at 23.21%, 22.53%, 11.2% and 24.86%, respectively, as shown in Table 1. The nutrient composition of the formulated experimental diets is provided in Table 3. The crude protein content of the four experimental diets remained almost similar, approximately 35%. In addition, the T_1_ (6.40%) diet exhibited the highest crude lipid content, whereas the control, T_0_ (5.49%) showed the lowest. Regarding crude fibre, T_3_ recorded the highest value, whereas both T_1_ and T_0_ had comparatively lower levels.

**TABLE 3:** Proximate composition of four experimental diets prepared with varying levels of papaya leaf meal (PLM) (% moisture basis).

Proximate composition (%)	Treatments			
	T_0_ 0% PLM	T_1_ 3% PLM	T_2_ 6% PLM	T_3_ 9% PLM
Moisture	11.38	12.03	11.24	11.41
Crude lipid	5.49	6.40	6.35	6.14
Crude protein	35.12	34.90	34.73	35.01
Ash	14.63	13.74	14.24	15.16
Crude fibre	4.20	4.58	4.85	5.06
Total	100	100	100	100

### 3.2 Growth Performance of Tilapia

The results from this study indicate significant differences in most of the fish growth parameters among the treatments (*p* < 0.05), as summarized in Table 4. The control group (T_0_), which had no PLM in the diet, generally recorded lower values compared to the PLM‐supplemented treatments. In particular, the T_3_ treatment, where 9% PLM was added, showed significantly (*p* < 0.05) higher values for mean weight gain (11.44 ± 0.48) compared to the other groups T_0_ (8.45 ± 1.01), T_1_ (9.07 ± 1.55), T_2_ (10.32 ± 0.10). Regarding specific growth rate (SGR), the highest value was 1.06 ± 0.02%/day observed in T_3_, whereas T_0_ had the lowest of 0.69 ± 0.07%/day. Similar results were observed for both protein efficiency ratio (PER) (1.28 ± 0.06) and feed conversion efficiency (FCE) (0.44 ± 0.02) in treatment T_3_ where 9% PLM was incorporated. Moreover, higher production (2.04 ± 0.02) was also reported from T_3_ compared to control (1.55 ± 0.17), followed by T_1_ (1.66 ± 0.12) and T_2_ (1.83 ± 0.12) (Table 4). Furthermore, better food conversion ratio (FCR) was observed in T_3_ where the value was significantly (*p* < 0.05) lower (2.23 ± 0.10) than the other treatments. The amount of protein intake and per cent survival rate didn't statistically vary (*p* > 0.05) among the groups and remained between 8.58 ± 0.58 to 8.96 ± 0.04 and 85.24 ± 3.30 to 88.09 ± 6.60 per cent, respectively.

**TABLE 4:** Growth performance, survival rate and production of *Oreochromis niloticus* fingerlings fed varying levels of papaya leaf meal (PLM)‐based diet for a period of 60 days.

Parameters	Treatments				*F* value	*p* value	Level of sig.
	T_0_ 0% PLM	T_1_ 3% PLM	T_2_ 6% PLM	T_3_ 9% PLM			
MIW (g)	14.73 ± 0.60	14.83 ± 1.06	15.10 ± 0.85	14.53 ± 0.27	1.157	0.384	NS
MFW (g)	22.45 ± 1.20^c^	23.90 ± 0.78^b^	24.60 ± 0.30^b^	26.47 ± 0.11^a^	15.491	0.001	**
MWG (g)	8.45 ± 1.01^b^	9.07 ± 1.55^b^	10.32 ± 0.10^ab^	11.44 ± 0.48^a^	5.770	0.021	**
(%) WG	57.44 ± 7.04^b^	61.82 ± 15.15^b^	68.54 ± 4.54^ab^	82.67 ± 2.07^a^	4.660	0.036	**
SGR (%/day)	0.69 ± 0.07^b^	0.81 ± 0.16^b^	0.81 ± 0.09^b^	1.06 ± 0.02^a^	5.399	0.025	**
FCR	2.93 ± 0.23^a^	2.77 ± 0.44^a^	2.48 ± 0.08^ab^	2.23 ± 0.10^b^	4.461	0.040	**
FCE	0.34 ± 0.02^b^	0.37 ± 0.06^b^	0.40 ± 0.01^ab^	0.44 ± 0.02^a^	5.198	0.028	**
PI	8.58 ± 0.58	8.60 ± 0.19	8.90 ± 0.29	8.96 ± 0.04	1.104	0.402	NS
PER	0.98 ± 0.07^b^	1.05 ± 0.17^b^	1.15 ± 0.04^ab^	1.28 ± 0.06^a^	4.978	0.031	**
Survival rate (%)	88.09 ± 6.60	85.24 ± 3.30	86.19 ± 8.23	86.19 ± 6.60	0.104	0.956	NS
Production (kg/m^3^/60 days)	1.55 ± 0.17^b^	1.66 ± 0.12^b^	1.83 ± 0.12^ab^	2.04 ± 0.02^a^	5.198	0.028	**

*Note*: Values are given with (±SD). The values in the same row having similar letter(s) do not differ significantly otherwise differ significantly (*p* < 0.05) as per DMRT (Duncan Multiple Range Test). ‘*’ indicates that significant at *p* < 0.05 and ‘**’ indicates that significant at *p* < 0.01. ‘NS’ indicates that not significant at *p* > 0.05.

Abbreviations: FCE, feed conversion efficiency; FCR, food conversion ratio; PER, protein efficiency ratio; SGR, specific growth rate; WG, weight gain.

### 3.3 Length–Weight Relationship

The regression analysis revealed a strong positive correlation between the length and weight of tilapia fingerlings fed with T_0_, T_1_, T_2_ and T_3_ diets, with correlation coefficients (*r*) of 0.94, 0.95, 0.96 and 0.96, respectively. The regression exponent (*b*) values for all treatments were less than 3 (T_0_ = 2.0549, T_1_ = 2.0526, T_2_ = 2.1286 and T_3_ = 2.1669), indicating a negative allometric growth pattern in the experimental fish. The regression graphs illustrating the length–weight relationship are shown in Figure 1.

**FIGURE 1:**
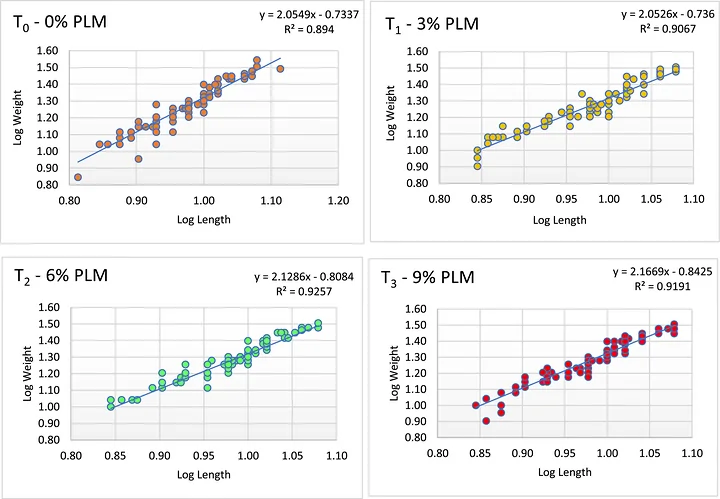
Length–weight relationship of tilapia fingerling fed T_0_, T_1,_ T_2_ and T_3_ diet. PLM, papaya leaf meal.

### 3.4 Nutritional Composition of Tilapia Fillet

The nutritional content of tilapia showed significant differences among the treatments (*p* < 0.05). The moisture content of fingerlings was recorded as 78.01% ± 0.01% in T_0_, 77.85% ± 0.03% in T_1_, 76.34% ± 0.02% in T_2_ and 77.56% ± 0.03% in T_3_. The highest crude protein content was observed in T_2_ (15.56% ± 0.04%), whereas the lowest was found in T_0_ (13.34% ± 0.01%). On the other hand, the highest crude lipid of tilapia was 2.34% ± 0.02% in T_3_, followed by 2.23% ± 0.03, 1.89% ± 0.04 and 1.67% ± 0.01%, in T_1_, T_2_ and T_0_, respectively (Table 5). However, fish fed 9% PLM (T_3_) represented higher ash content (3.67 ± 0.01) compared to the others (T_0_—3.55 ± 0.05, T_1_—3.34 ± 0.01, T_2_—2.90 ± 0.01).

**TABLE 5:** Proximate composition of tilapia fingerlings fed varying levels of papaya leaf meal (PLM) for a period of 60 days (% moisture basis).

Nutritional component (%)	Treatments				*F* value	*p* value	Level of sig.
	T_0_ 0% PLM	T_1_ 3% PLM	T_2_ 6% PLM	T_3_ 9% PLM			
Moisture	78.01 ± 0.01^a^	77.85 ± 0.03^ab^	76.34 ± 0.02^b^	77.56 ± 0.03^ab^	3007.29	0.01	**
Crude protein	13.34 ± 0.01^b^	14.73 ± 0.01^ab^	15.56 ± 0.04^a^	14.21± 0.02^ab^	4726.90	0.01	**
Crude lipid	1.67 ± 0.01^b^	2.23 ± 0.03^ab^	1.89 ± 0.04^b^	2.34 ± 0.02^a^	380.367	0.01	**
Crude fibre	1.74 ± 0.02^a^	1.54 ± 0.01^ab^	1.45 ± 0.05^b^	1.64 ± 0.02^ab^	55.38	0.01	**
Ash	3.55 ± 0.05^ab^	3.34 ± 0.01^b^	2.90 ± 0.01^c^	3.67 ± 0.01^a^	491.57	0.01	**
Carbohydrate	1.69 ± 0.02^ab^	0.31 ± 0.02^c^	1.86 ± 0.03^a^	0.58 ± 0.02^b^	4006.42	0.01	**

*Note*: Values are given with (±SD). The values in the same row having similar letter(s) do not differ significantly otherwise differ significantly (*p* < 0.05) as per DMRT (Duncan Multiple Range Test). ‘*’ indicates that significant at *p* < 0.05 and ‘**’ indicates that significant at *p* < 0.01. ‘NS’ indicates that not significant at *p* > 0.05.

### 3.5 Haematological Parameters

Haematological assessments were carried out to evaluate the physiological responses of Nile tilapia to varying levels of dietary PLM, as illustrated in Figure 2. A significant variation in RBC counts was observed among the treatments (*p* < 0.05), with the highest value recorded in T_3_ (3.10 ± 0.95 × 10^6^/mm^3^) and the lowest in T_0_ (2.20 ± 0.87 × 10^6^/mm^3^) followed by T_1_ (2.70 ± 1.33 × 10^6^/mm^3^) and T_2_ (2.60 ± 1.46 × 10^6^/mm^3^). A similar trend was observed for WBC counts, with T_3_ showing significantly higher levels (132.98 ± 2.26 × 10^3^/mm^3^) compared to T_0_ (93.83 ± 2.44 × 10^3^/mm^3^), T_1_ (114.79 ± 2.8 × 10^3^/mm^3^) and T_2_ (103.351 ± 3.08 × 10^3^/mm^3^). Haemoglobin (Hb) concentrations also increased significantly with higher PLM inclusion (*p* < 0.05), ranging from 9.68 ± 0.61 g/dL in T_0_ to 12.15 ± 0.68 g/dL in T_3_. In contrast, higher glucose (Glc) reading was observed in T_0_ (113.33 ± 3.80 mg/dL) followed by T_2_ (105.67 ± 3.88 mg/dL), T_1_ (92 ± 2.5) and T_3_ (90 ± 2.3), whereas MCH levels did not differ significantly among treatments.

**FIGURE 2:**
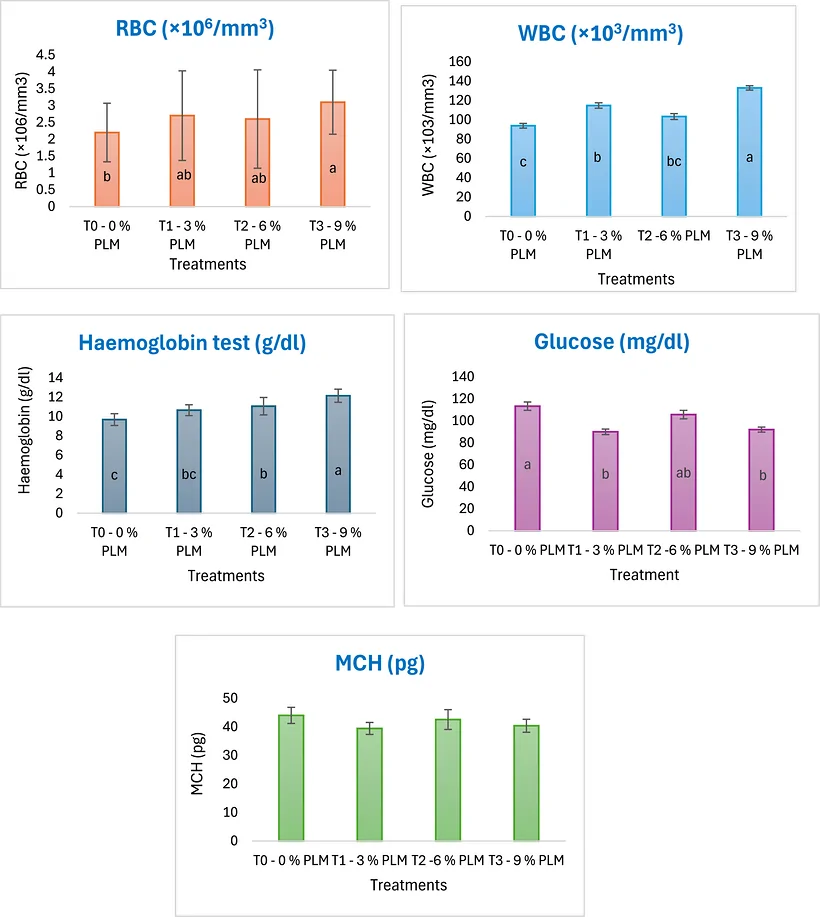
RBC, WBC, haemoglobin, glucose and MCH of Nile tilapia fed PLM‐based diet for a period of 60 days. MCH, mean corpuscular haemoglobin; PLM, papaya leaf meal; RBC, red blood cell; WBC, white blood cell.

### 3.6 Water Quality Parameters

Throughout the study, key water quality parameters, including temperature, DO, pH, NH_3_, NO_3_, TDS and EC remained relatively stable across all treatments, with no statistically significant variations observed (*p* > 0.05), as shown in Table 6. All measured values stayed within optimal ranges for tilapia culture. However, ammonia levels showed a slight upward trend toward the conclusion of the experiment.

**TABLE 6:** Water quality parameters of tilapia fed papaya leaf meal (PLM)‐based diet for a period of 60 days.

Parameters	Treatments				*F* value	*p* value	Level of sig.
	T_0_ 0% PLM	T_1_ 3% PLM	T_2_ 6% PLM	T_3_ 9% PLM			
Temperature (°C)	22.80 ± 0.70	22.80 ± 0.63	22.80 ± 0.63	22.80 ± 0.63	0.006	0.999	NS
pH	7.20 ± 0.29	6.95 ± 0.43	7.34 ± 0.20	7.10 ± 0.17	1.317	0.314	NS
NH_3_	0.18 ± 0.13	0.18 ± 0.13	0.18 ± 0.13	0.23 ± 0.17	1.060	0.402	NS
NO_3_	5 ± 0.02	5 ± 0.01	5 ± 0.01	6.25 ± 0.01	0.558	0.614	NS
DO (ppm)	4.80 ± 0.57	4.55 ± 0.57	5.00 ± 0.57	5.20 ± 0.57	0.005	0.890	NS
EC	400 ± 22.60	378.50 ± 19.82	404.42 ± 20.67	395.33 ± 25.77	0.557	0.654	NS
TDS (ppm)	200 ± 16.97	191.17 ± 13.63	201.50 ± 15.06	197.68 ± 13.30	0.324	0.808	NS

*Note*: Values are given with (±SD). The values in the same row having similar letter(s) do not differ significantly otherwise differ significantly (*p < *0.05) as per DMRT (Duncan Multiple Range Test). ‘NS’ indicates that not significant at *p *> 0.05.

Abbreviation: TDS, total dissolved solids; DO, Dissolved oxygen; EC, Electrical conductivity.

## 4 Discussion

### 4.1 Growth Performance

The aim of this experiment was to identify an easily accessible and nutrient‐rich feed ingredient with high essential contents to enhance fish growth performance. In this study, Nile tilapia (*O. niloticus*) fingerlings were fed diets containing different levels of PLM. The study showed that incorporating PLM into the diet improved nutrient utilization. The growth parameters of fish fed PLM‐based diets demonstrated significant difference to those of the control group. These findings are consistent with previous studies. Tewari et al. (2018) found that 2.5% papaya leaf inclusion in diet significantly (*p* < 0.05) enhanced the growth of *Cyprinus carpio* compared to control. Additionally, Hamid et al. (2022) observed notable improvements in weight gain, SGR and feed efficiency in red hybrid tilapia with the inclusion of papaya leaf extract, recommending its use as a feed additive to promote fry growth, which aligns to the present study.

In the present study, per cent weight gain, SGR and PER of the T_3_ treatments were statistically significant (*p* < 0.05) than those of the control, indicating a satisfactory growth trend with increased PLM inclusion. These findings align with Munguti et al. (2014) and Mic Salomiya et al. (2023), who reported improved per cent weight gain and SGR when fed diets supplemented with crude papain from papaya leaves to Nile tilapia (*O. niloticus*) and *L. rohita*, respectively. In addition, Jamal et al. (2025) found that papaya seed supplementation improved SGR and PER in *C. carpio*, particularly at inclusion levels of 6 and 7 g/kg, whereas other studies recorded (Radwan et al. 2023: 2% papaya seed meal, Hamid et al. 2022: 2% PLM and Ugonna et al. 2018: 4% papaya seed extract) better growth performance to Nile tilapia fingerling feeding lower level of papaya leaf and seed extracts. The current results are slightly higher than the previous. This could result due to the higher amount of papain enzyme present in leaf meal, helping in protein digestion, that might have a positive effect on growth performance when incorporated into a certain percentage. Furthermore, feed consumption showed variation across treatments, suggesting that the fish's appetite was affected by the taste of diets containing different levels of *C. papaya* leaf meal. In the current research, lower FCR was reported from treatment T_3_ where 9% PLM was added that showed a similarity with the result of Mic Salomiya et al. (2023) who found a better FCR in rohu (*L. rohita*) fed with PLM.

### 4.2 Carcass Composition of Tilapia

The crude protein and lipid content were notably higher in the T_2_ and T_3_ diets than in the control, with the PER also being greater in the T_3_ treatment. This indicates that the protein source might have improved the fish's capacity to utilize essential amino acids. The present study finds similarity with the study of Matoria et al. (2023) who also reported higher percentage of crude protein (15.84 ± 0.032) in Nile tilapia carcass composition fed with 8% PLM diet. Another observation was made by Helmiati et al. (2020), who found variations in protein content in red tilapia (*Oreochromis* sp.) fed a diet with fermented moringa leaves. Although limited information is available on the proximate composition of tilapia fed varying levels of PLM, this study found a significant difference compared to the control.

### 4.3 Length–Weight Relationship

The length–weight relationship provides insights into a species’ growth and overall well‐being (Okgerman 2005; Thulasitha and Sivashanthini 2012). In this study, Nile tilapia fingerlings exhibited negative allometric growth; however, there was a substantial positive correlation (*r* = 0.94–0.96) between the fish's length and weight during the experimental period when fed the tested diets. Tewari et al. (2018) observed isometric growth in fish fed a papain‐supplemented diet, whereas Ngodhe and Owuor‐JB (2019) reported positive allometric growth in both wild and cage‐reared Nile tilapia. The previous studies represent higher value than the present one. Variations in (*b*) values can be explained by a combination of one or more factors, including number of specimens examined, seasonal effect, habitat and degree of stomach fullness (Karrar et al. 2016). This suggests that the papain enzyme might not have any negative impact on growth response in Nile tilapia. The differences from the current study are likely due to variations in culture duration, rearing conditions, feed composition and environmental factors such as water temperature.

### 4.4 Haematological Performance

The haematological assessment in the present study revealed that RBCs and WBCs count and haemoglobin (Hb) levels increased as the inclusion of PLM in the diet increased. These results suggest that the fish may have stronger immune responses, leading to improved blood properties. Additionally, the higher concentration of micronutrients in PLM could have contributed to the increased Hb levels. Furthermore, the glucose level of tilapia is better in higher inclusion level of PLM‐based diet. However, there were no significant differences in MCH levels compared to the control group. Olusola and Nwokike (2018) reported significant differences (*p* < 0.05) in Hb, RBC and WBC counts across dietary groups, with RBC and WBC levels increasing as the papaya leaf‐based diet inclusion increased compared to the control. Likewise, Oparaku et al. (2024) and Radwan et al. (2023) observed significant increases in RBC and Hb levels in fish exposed to *C. papaya*, aligning with the findings of this study. Similar findings were reported by several studies that observed higher RBC counts and Hb levels in *C. gariepinus* (Ayoola et al. 2013) and *Barbonymus gonionotus* (Farhad et al. 2023) fed a moringa leaf‐based diet compared to the control group. In contrast, Hamid et al. (2022) and Solomon et al. (2017) found no significant differences in RBC and Hb levels compared to the control, except for MCH when fed *C. papaya*‐based diet to Nile tilapia.

## 5 Conclusion

This study demonstrates that PLM has potential to be used as a growth enhancer in Nile tilapia diets because it improved their growth performance and yielded better economic benefits by using unconventional feed ingredients. The blood parameters, such as RBCs, WBCs and Hb, were notably higher in fish feeding 6% and 9% PLM, whereas the glucose content remained between optimal range in 3% and 9% based diets, showing significant improvement across all experimental diets. The length–weight relationship also represents positive allometric growth pattern of tilapia fingerling. This suggests that PLM positively influenced the well‐being of Nile tilapia compared to the diet without PLM. Therefore, fish farmers can consider supplementing fish diets with papaya leaves up to 9%, as they are readily available and can serve as an alternative protein source.

## Author Contributions

All authors collaborated in formulating and refining the study's conception and design. Farhabun Binte Farhad thoroughly conducted and monitored the experiment, data collection, curated the data, writing and reviewing the draft. Farzana Yasmin Ritu and Moupia Mustafi contributed to data collection, data curation and writing the draft manuscript. Shaharior Hashem contributed to data curation, drafting and provided critical input for writing, reviewing and editing. Md. Abdus Salam led the conceptualization and design of the study and authored the original manuscript.

## Ethics Statement

The authors confirm that the ethical policies of the journal, as noted on the journal's author guidelines page, have been adhered to and the appropriate ethical review committee approval has been received. The Bangladesh Agricultural University's guidelines for the Care and Use of Laboratory Animals were followed.

## Conflicts of Interest

The authors declare no conflicts of interest.

## Data Availability

The data underlying the results of this study can be obtained from the corresponding author upon a reasonable request.
